# NKG2A‐checkpoint inhibition and its blockade critically depends on peptides presented by its ligand HLA‐E

**DOI:** 10.1111/imm.13515

**Published:** 2022-06-06

**Authors:** Claire Battin, Gabriel Kaufmann, Judith Leitner, Joshua Tobias, Ursula Wiedermann, Alexander Rölle, Marten Meyer, Frank Momburg, Peter Steinberger

**Affiliations:** ^1^ Division of Immune Receptors and T Cell Activation, Center for Pathophysiology, Infectiology and Immunology Medical University of Vienna Vienna Austria; ^2^ Institute of Specific Prophylaxis and Tropical Medicine, Center for Pathophysiology, Infectiology and Immunology Medical University of Vienna Vienna Austria; ^3^ Clinical Cooperation Unit “Applied Tumor Immunity” German Cancer Research Center Heidelberg Germany; ^4^ Department of Medical Oncology, National Center for Tumor Diseases University Hospital Heidelberg Heidelberg Germany; ^5^ Antigen Presentation and T/NK Cell Activation Group DKFZ Heidelberg Germany

**Keywords:** checkpoint inhibitor, monalizumab, NKG2A

## Abstract

NKG2A has emerged as a new immunotherapy target and its blockade with the novel immune checkpoint inhibitor (ICI) monalizumab can boost both NK cell and CD8^+^ T cell responses. NKG2A forms heterodimers with CD94 and binds to the human non‐classical MHC class I molecule HLA‐E. HLA‐E forms complexes with a limited set of peptides mainly derived from the leader sequences of the classical MHC class I molecules (HLA‐A, HLA‐B and HLA‐C) and the non‐classical class I paralogue HLA‐G, and it is well established that the interaction between CD94/NKG2x receptors and its ligand HLA‐E is peptide‐sensitive. Here, we have evaluated peptide dependence of NKG2A‐mediated inhibition and the efficiency of interference by monalizumab in a transcriptional T cell reporter system. NKG2A inhibition was mediated by cell‐expressed HLA‐E molecules stably presenting disulfate‐trapped peptide ligands. We show that different HLA‐class I leader peptides mediate varying levels of inhibition. We have used NKG2A/NKG2C chimeric receptors to map the binding site of NKG2A and NKG2C blocking antibodies. Furthermore, we determined the functional EC_50_ values of blocking NKG2A antibodies and show that they greatly depend on the HLA‐leader peptide presented by HLA‐E. Monalizumab was less effective in augmenting NK cell‐mediated killing of target cells displaying HLA‐G peptide on HLA‐E, than cells expressing HLA‐E complexed with HLA‐A, HLA‐B and HLA‐C peptides. Our results indicate that peptides displayed by HLA‐E molecules on tumour cells might influence the effectivity of NKG2A‐ICI therapy and potentially suggest novel approaches for patient stratification, for example, based on tumoral HLA‐G levels.

## Graphical abstract

NKG2A has emerged as a new immunotherapy target and its blockade with the novel immune checkpoint inhibitor monalizumab can boost both NK cell and CD8^+^ T cell responses. The interaction between CD94/NKG2x receptors and its ligand HLA‐E is peptide‐sensitive. The peptides displayed by HLA‐E might influence the effectivity of NKG2A immune checkpoint therapy.

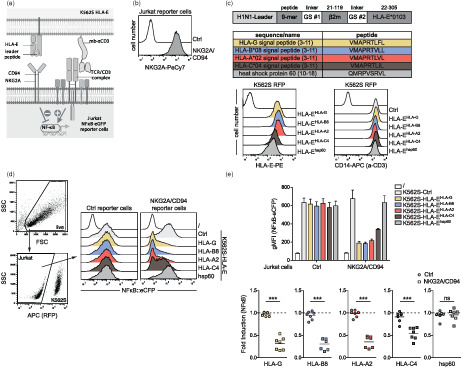

**Abbreviations**
eGFP: enhanced green fluorescent protein
hsp60: heat shock protein 60
ICI: immune checkpoint inhibitor
RFP: red‐fluorescent protein
SCT: single chain trimers

## INTRODUCTION

The role of immune checkpoints in preventing antitumour immunity has led to a paradigm shift in cancer immunotherapy [1, 2]. Therapeutic monoclonal antibodies targeting inhibitory receptors like PD‐1 and CTLA‐4 can revert T cell inhibition, thereby improving antitumour responses. In the last decades, immune checkpoint inhibitors (ICIs), like nivolumab or ipilimumab, have revolutionized cancer treatment [3]. NKG2A (KLRC1; CD159a) has emerged as a new checkpoint receptor and its blockade has shown promising results in boosting NK cell and CD8^+^ T cell effector functions in humans [4]. NKG2A, part of the NKG2x family of proteins, is a C‐type lectin and dimerizes with CD94. It is expressed at different levels on the cell surface of CD56^high^ NK cells, NKT cells and cytotoxic CD8^+^ T cells. NKG2A harbours intracellular tyrosine‐based inhibitory motifs, which are phosphorylated upon receptor engagement. This results in the recruitment of phosphatases, which transduce inhibitory signals into immune cells [5]. A close relative of NKG2A is the activating receptor NKG2C which associates with CD94 and DAP12 and transmits stimulatory signals via the tyrosine‐based activating motifs [6].

Increased NKG2A expression was observed on cytotoxic CD8^+^ T cells and NK cells in the tumour bed or surrounding tissue [4, 7, 8]. T cell receptor triggering induces NKG2A expression in tumour‐specific CD8+ T cells [9]. Recently, Andre et al. proposed monalizumab, a humanized anti‐NKG2A IgG4 blocking mAb, as a novel ICI that promotes T cell‐ and NK cell‐mediated antitumour immunity [4, 10]. It has been reported that combination therapy targeting the PD‐1/PD‐L1 axis and NKG2A (with monalizumab), enhanced NK cell activity by increasing degranulation and IFN‐γ production, and rescued CD8 T cell function in various cancer models [4]. In tumour‐bearing mice, peptide vaccination followed by NKG2A blocking resulted in enhanced CD8 T cell immunity and a reduction in tumour progression [9]. Moreover, in patients with head and neck carcinomas combined therapy of cetuximab and monalizumab promoted ADCC and led to enhanced antitumour responses [4].

The NKG2A and NKG2C receptors bind to the non‐classical MHC class I molecule HLA‐E, which is broadly expressed at low levels in most tissues but is abundant in immune‐privileged sites [11, 12]. In contrast to the other MHC molecules, HLA‐E mainly presents a limited set of peptides that are derived from leader sequences of classical MHC class I molecules (HLA‐A, HLA‐B and HLA‐C) and non‐classical class I paralogue HLA‐G [13, 14]. HLA‐E can also present peptides from stress‐induced proteins like heat shock protein 60 (hsp60) [15]. While classical MHC molecules are frequently lost by tumours to escape T cell control, various studies report high HLA‐E protein levels in cancer compared to healthy controls [16, 17, 18, 19]. Importantly, high expression of HLA‐E is related to poorer prognosis in cancer patients [20, 21]. The propensity for HLA‐E expression in malignant cells is still not fully understood, and it has been suggested that tumours may upregulate HLA‐E and HLA‐G to escape killing by immune cells [22]. NKG2A interacts with significantly higher affinities with HLA‐E‐peptide complexes than the activating receptor NKG2C [23, 24]. Recently, Rölle et al. have demonstrated that NKG2C expressing NK cells discriminate between peptides (MHC class I leader sequences) that are displayed in the HLA‐E molecule [25]. Moreover, it was shown that a patient's MHC class I genotype plays indeed an important role in checkpoint immunotherapy which may relate to MHC class I peptide presentation by HLA‐E and its interaction with the inhibitory receptor NKG2A [26].

Here, we used a fluorescent NFκB transcriptional reporter system in conjunction with cells expressing stabilized HLA‐E‐peptide complexes to show that minor differences in the leader sequences from MHC class I molecules presented by the HLA‐E ligand result in distinct degrees of NKG2A inhibition. Chimeric NKG2A/NKG2C receptors were used to identify the epitopes of monalizumab and other NKG2A blocking antibodies. In line with data from our reporter model, we found that the protection of HLA‐E expressing target cells from NK‐mediated lysis critically depends on the presented peptides. Our results highlight the crucial importance of these peptides presented by HLA‐E, but also how they impact the capacity of checkpoint inhibitors such as monalizumab to efficiently block the NKG2A/HLA‐E inhibitory pathway. The formation of high‐affinity HLA‐E‐peptide complexes could potentially contribute to resistance to ICI therapies targeting NKG2A.

## MATERIAL AND METHODS

### Sample collection

The study with primary human cells was approved by the ethics committee of the Medical University of Vienna (1183/2016). Procedures with human material were performed by ethical standards of the ethics committee and the Helsinki Declaration of 1964 and its later amendments. Blood samples were collected from healthy donors and PBMCs were isolated from heparinized whole blood samples with standard gradient density centrifugation using Lymphoprep solution (Technoclone).

### Cell culture, antibodies and flow cytometry

The Jurkat (JE6.1) cell line and the human erythroleukemia K562 cell line were derived from in house stocks. The reporter cell line, based on the JE6.1 cell line, was generated as previously described [27]. The stimulator cells (K562S), which are based on the K562 cell line, were transduced to stably express a membrane‐bound human CD3 Ab single‐chain fragment (CD14 stem) and have been described in detail [28]. All cell lines within this study were cultured in RPMI1640 supplemented with 10% FBS, penicillin (100 U/ml) and streptomycin (100 μg/ml) (all from Sigma‐Aldrich). All cells were routinely tested for mycoplasma contaminations [29]. To assess cell surface expression the following antibodies were used: NKG2A‐PeCy7 (S19004C), NKG2C‐PE (S19005E), CD14‐APC (M5E2), CD3‐APC (UCHT1), CD4‐BV421 (OKT4), CD8‐PerCP (HIT8α), CD56‐PE (5.1H11), CD56‐BV510 (5.1H11), CD25‐PeCy7 (M‐A251) and HLA‐E‐PE (3D12) all from Biolegend. Anti‐Human KLRC1 therapeutic antibody (Monalizumab) was purchased from Creative Biolabs. Human NKG2A antibody (clone REA110) was from Miltenyi Biotec and clone Z199 was from Beckman Coulter Inc. Human NKG2A antibody (131411) and NKG2C antibody (134522) were from R&D systems. Unconjugated purified human HLA‐E (3D12) was purchased from Biolegend. Surface expression of unconjugated antibodies and HLA‐E‐Fc single chain trimers (SCT) were detected with a goat anti‐mouse‐PE/APC IgG (Fc‐specific) or goat anti‐human‐PE/APC IgG (Fc‐specific) antibody (Jackson ImmunoResearch), respectively. The Strep‐tag was detected by using the monoclonal mouse NWSHPQFEK‐Tag (biotin) antibody (GeneScript) in conjunction with a streptavidin‐PE (Biolegend). To exclude K562 cells from reporter cells, they were transduced to constantly express a red‐fluorescent protein (RFP). For cell–cell interaction studies, K562 cells were transduced with NKG2A/CD94 as well as a green fluorescent protein (eGFP). Flow cytometry analysis was performed using FACSCalibur or LSRFortessa flow cytometers (BD Bioscience) according to guidelines. FlowJo software (version 10.4.1; Tree Star) was used for flow cytometry data analysis.

### Lentiviral transduction

Reporter cells were engineered to express NKG2A (UniProt P26715‐1) or NKG2C (UniProt P26717‐1) with CD94 (Uniprot Q13241) or DAP12 (Uniprot O43914), respectively, via lentiviral transduction (pHR‐SIN‐BX_IRES‐Emerald) followed by puromycin and blasticidin selection [30]. The chimeric constructs NKG2A‐C #1 (aa 1–132 of UniProt P26715‐1 and aa 131–231 of UniProt P26717‐1), NKG2A‐C #2 (aa 1–180 of UniProt P26715‐1 and aa 179–231 of UniProt P26717‐1), NKG2A‐C #3 (aa 1–206 of UniProt P26715‐1 and aa 205–231 of UniProt P26717‐1) and NKG2C_mut_ (I187M, K195E) were cloned and lentivirally transduced into the Jurkat cells. Membrane‐bound versions of peptide‐β2m‐HLA‐E SCT were generated based on previously described SCT‐immunoglobulin fusion proteins [25]. A pBluescript KS II(+) vector encoding the influenza virus hemagglutinin H1N1 leader (M1‐A17), a 9‐mer peptide of choice (e.g., VMAPRTLFL derived from the HLA‐G leader sequence), a linker containing a cysteine (GCGSGGGGAPGSGGGS), a leader‐less β2‐microglobulin (GenBank: BC064910, I21‐M119), a second linker (RSASGGGGSGGGGSGGGGSASGGG), and the leader‐less HLA‐E (allele HLA‐E*0103; NM_005516, G22‐L358 with a Y84 to C mutation to form the disulfide trap with the cysteine in the first linker) was a gift of Frank Momburg, German Cancer Research Center, Heidelberg [25]. Sequences encoding β2m‐HLA‐E molecules presenting the different 9‐mer peptide leader sequences (HLA‐G, VMAPRTLFL; HLA‐C*04:01, VMAPRTLIL; HLA‐A*02:01, VMAPRTLVL; HLA‐B*08:01, VMAPRTVLL and hsp60, QMRPVSRVL) were cloned into the pHR lentiviral vector. K562S RFP cells were lentivirally transduced to express peptide‐β2m‐HLA‐E on the cell surface.

### Binding assays

NKG2A/CD94 as well as NKG2C/CD94/DAP12 expressing reporter cells (1 × 10^5^) were incubated for 30 min at 4°C  with monalizumab, REA110, Z199, 131411 and 134522 at final concentrations of 10 μg/ml, 3.16 μg/ml, 1 μg/ml, 316 ng/ml, 100 ng/ml, 31.6 ng/ml, 10 ng/ml, 3.16 ng/ml, 1 ng/ml, 316 pg/ml and 100 pg/ml, respectively. Binding was detected with an APC‐conjugated goat anti‐mouse IgG (Fc‐specific) and goat anti‐human IgG (Fc‐specific) (Jackson ImmunoResearch) via flow cytometry. Human β2m‐HLA‐E‐mIgG2a‐Fc‐StrepTag fusion proteins (SCT) presenting peptides HLA‐G, HLA‐C4 and hsp60 have been previously described [25]. Control, NKG2A or NKG2C expressing reporter cells were incubated with the HLA‐E SCT‐Fc molecules and binding was analysed using a PE‐conjugated goat anti‐mouse (Fc‐specific) antibody. For cell conjugation assays, K562 cells were first transduced to express either eGFP or RFP. Subsequently, NKG2A/CD94 was expressed in K562 eGFP, while HLA‐E^peptide^ was introduced into K562 RFP cells; 4 × 10^4^ (for each cell line, in full RPMI medium) were mixed in the presence or absence of monalizumab (10 and 3 μg/ml). Cells were incubated for 4 h at 37°C with 5% CO_2,_ then gently mixed and cell–cell conjugates were measured via flow cytometry.

### Reporter assays

For the functional in vitro assays, NKG2A or NKG2C expressing reporter cells (5 × 10^4^ cells/well) were co‐cultured with control K56S and K562S HLA‐E^peptide^ expressing cells (2 × 10^4^ cells/well) in the presence or absence of NKG2A antibodies (31.6 μg/ml, 10 μg/ml, 3.16 μg/ml, 1 μg/ml, 316 ng/ml, 100 ng/ml, 31.6 ng/ml and 10 ng/ml) or HLA‐E antibody (3D12; 5 μg/ml) for 24 h at 37°C with 5% CO_2_. Jurkat cells were pre‐incubated with the antibodies for 15 min at RT before K562S cells were added. Subsequently, cells were harvested and reporter activation (eCFP or eGFP expression) was measured via flow cytometry [27, 31]. The geometric mean of fluorescence intensity of viable reporter cells (RFP negative) was used for further analysis. For some experiments, reporter gene induction in response to K562 HLA‐E^peptide^ stimulation was normalized to control K562‐stimulated reporter cells as indicated and expressed as fold induction. To calculate the functional EC_50_ values, three (monalizumab) and two (Z199) independent reporter stimulation experiments were performed in duplicate. For each experiment, reporter gene expression with or without antibody was normalized in the presence of K562S HLA‐E^peptide^ to control K562S in the respective condition and depicted as fold induction. The EC_50_ values and the 95% confidence intervals (CIs) were determined using the two‐parameter non‐linear regression by putting the top values to 1 and the bottom to the values obtained with the lowest antibody concentration (10 ng/ml) (GraphPad Prism V5.0b).

### CFSE proliferation assays

PBMCs were labelled with 1 μM (final in 1x PBS) CFSE solution (C34554; Molecular Probes) as previously described [32]. CFSE‐labelled PBMCs were pre‐incubated with monalizumab (5 μg/ml) for 15 min at room temperature (RT). T cell stimulator cells (K562S cells) were pre‐treated with mitomycin C (final concentration 20 μg/ml; Carl Roth) for 30 min at 37°C. Subsequently, they were washed three times with 1x PBS. Then, 1 × 10^4^ control K562S, K562S‐HLA‐E^HLA‐G^, K562S‐HLA‐E^HLA‐B8^, K562S‐HLA‐E^HLA‐A2^, K562S‐HLA‐E^HLA‐C4^ and K562S‐HLA‐E^hsp60^ were added to 1 × 10^5^ CFSE‐labelled PBMCs. After 5 days, PBMCs were harvested and stained for CD4, CD8 and CD56. CFSE^low^ expression was analysed on CD8^−^CD4^−^CD56^+^ cell subsets. A single data point represents one donor.

### Cytotoxicity assays

For cellular cytotoxicity assays, isolated PBMCs were cultured for 7–10 days in MEM medium supplemented with 3% human serum, 200 U/ml IL‐2 and 150 U/ml IL‐15. Cells were stained for CD3, CD4, CD8, CD56, NKG2A and NKG2C expression prior activation and on days 7–10. Then, 1 × 10^5^ activated PBMCs were co‐cultured with 1 × 10^4^ K562‐HLA‐E^peptide^ (w/o anti‐CD3) (E:T = 10:1)) in the presence or absence of 5 μg/ml monalizumab for 4 h at 37°C in 5% CO_2_. NK cell expansion samples were pre‐incubated with monalizumab for 15 min at RT. To determine specific cell lysis counting beads (123count eBeads™ Counting Beads) were added and an equal amount for each sample was acquired. The amount of K562 cells was normalized to acquired beads in each sample. The percentage of specific lysis was calculated using the following formula:

$$\%\;\text{cell lysis}=100-\frac{\left(100\times \text{amount of}\;K562\;\text{(in the presence of effector cells)}\right)}{\text{amount of}\;K562\;\text{cells (in the absence of effector cells)}}$$

### Statistics

**FIGURE 1:**
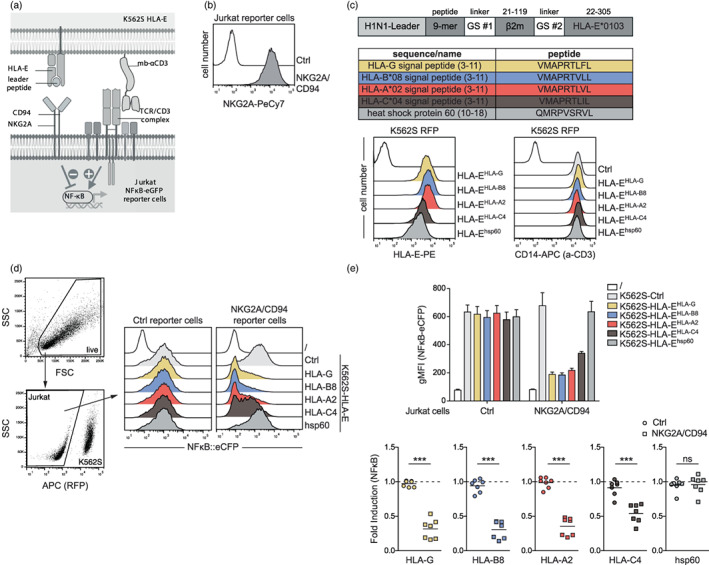
Use of a reporter cell system to evaluate NKG2A/CD94 inhibition and its interaction with peptide‐HLA‐E complexes. (a) Schematic showing the interaction between NKG2A and the TCR/CD3 complex on the Jurkat reporter cells with HLA‐E‐peptide complexes and membrane‐bound anti‐CD3‐scFv on the T cell stimulator cells (K562S). (b) Surface expression of NKG2A on control and NKG2A/CD94 expressing reporter cells. (c) Schematic representation of a construct harbouring the sequences of the H1N1 leader, a 9‐mer peptide, GS‐linker (#1), β_2_‐microglobulin, GS‐linker (#2) and HLA‐E*0103. In the table, the five different 9‐mer peptides used in this study are depicted. Flow cytometric analysis of T cell stimulator cells (K562S RFP). Left panel; HLA‐E expression on control K562S cells (open histogram) and K562S transduced to express peptide‐HLA‐E complexes (filled histograms). Right panel; expression of membrane‐bound anti‐CD3 Ab fragment (CD14‐stem) on K562 cells (open histogram) and K562S (with and without HLA‐E) cells (filled histograms). (d) Gating strategy of one representative experiment of control reporter cells and NKG2A/CD94 reporter cells stimulated with control K562S and K562S‐HLA‐E^HLA‐G^, K562S‐HLA‐E^HLA‐B8^, K562S‐HLA‐E^HLA‐A2^, K562S‐HLA‐E^HLA‐C4^ and K562S‐HLA‐E^hsp60^. Open histogram: unstimulated cells. NFκB‐eCFP expression was measured via flow cytometry. (e) Control and NKG2A/CD94 expressing reporter cells were stimulated with the indicated K562S cells. Reporter activation is shown as the geometric mean of fluorescence intensity (gMFI) of NFκB activation. Lower panel: reporter activation is shown as fold induction (gMFI of K562S‐HLA‐E^peptide^‐stimulated cells/gMFI control K562S‐stimulated cells). Results of seven independent experiments performed in duplicates are depicted. For statistical evaluation, a two‐way ANOVA followed by Bonferroni post hoc test was performed (***p ≤ 0.001; **p ≤ 0.01; *p ≤ 0.05; ^ns^ *p* > 0.05).

For experiments with NKG2A reporter cells (Figure 1e) and primary NK cells evaluating cytotoxicity (Figure 6d), a two‐way ANOVA followed by a Bonferroni post hoc test was performed. For experiments analysing the proliferation of NK cells (Figure 6c), a one‐way ANOVA followed by Dunn's multiple comparison test was performed. Statistical calculations were performed using GraphPad Prism (levels of significance were categorized as follows: ns, not significant *p* > 0.05; *p ≤ 0.05; **p ≤ 0.01; ***p ≤ 0.001).

## RESULTS

Peptides presented by HLA‐E have been described to have an impact on its interaction with the NKG2A/CD94 receptor [24, 26]. Here, we used a reporter platform to assess inhibitory signalling upon engagement of NKG2A with different HLA‐E peptide complexes. This system is based on the human T cell line Jurkat JE6.1, which harbours NFκB‐responsive elements coupled to eCFP or eGFP. NKG2A and CD94 were co‐expressed on our reporter cells, which are endogenously devoid of this receptor (Figure 1a,b). To assess NKG2A inhibition in the T cell reporter cells, we used K562‐based stimulator cells (K562S) that express a membrane‐bound anti‐CD3 Ab fragment, which activates the reporter cells by engaging the TCR‐CD3 complex [28] (Figure 1a). To analyse the interaction of different HLA‐E‐peptide complexes with NKG2A, we generated K562S stimulator cells expressing trimeric HLA‐E single chain constructs encoding the ectodomain of the HLA‐E heavy chain, β_2_‐microglobulin, and a disulfide‐trapped HLA‐E peptide ligand (Figure 1c). We designed five different HLA‐E constructs presenting the following peptides: HLA‐G signal peptide (3–11; VMAPRTLFL), HLA‐B*08 signal peptide (3–11; VMAPRTVLL), HLA‐A*02 signal peptide (3–11; VMAPRTLVL), HLA‐C*04 signal peptide (3–11; VMAPRTLIL) and hsp60 (10–18; QMRPVSRVL) [25] (Figure 1c). Co‐culture experiments with reporter cells expressing NKG2A/CD94 and control K562S or K562S presenting different HLA‐E‐peptides complexes were performed (Figure 1d,e). Stimulation of control reporter cells with K562S expressing various HLA‐E molecules or with K562S control cells led to similar NFκB activation (reporter gene expression) (Figure 1d,e). By contrast, we observed a significant reduction of NFκB‐activation when NKG2A/CD94 expressing reporter cells were stimulated with K562S‐HLA‐E^HLA‐G^, K562S‐HLA‐E^HLA‐B8^, K562S‐HLA‐E^HLA‐A2^ and K562S‐HLA‐E^HLA‐C4^ in comparison to K562S control cells. Furthermore, the interaction of NKG2A/CD94 with K562S‐HLA‐E^hsp60^ showed similar NFκB‐activation as control K562S indicating that HLA‐E^hsp60^ cannot functionally engage NKG2A/CD94. Moreover, these data clearly show that HLA‐E^HLA‐G^ has a higher capacity to trigger NKG2A/CD94 than HLA‐E complexed with the HLA‐C4 signal peptide (HLA‐E^HLA‐C4^), but also that HLA‐E complexed with signal peptides derived from HLA‐A2 and HLA‐B8 can engage NKG2A/CD94 with high affinity. Binding studies with Fc‐tagged HLA‐E molecules (HLA‐E‐SCT‐Fc; [25]) presenting HLA‐G, HLA‐C4 or hsp60 derived peptides to NKG2A expressing cells corroborated our functional data (Figure S1). HLA‐E^HLA‐G^ complexes strongly bound to NKG2A/CD94 expressing cells, whereas binding signals with HLA‐E^HLA‐C4^‐Fc were much weaker. HLA‐E^hsp60^‐Fc did not bind to NKG2A/CD94 (Figure S1). Overall, these results show that our T cell reporter platform represents a valuable tool to study NKG2A/CD94 signalling and its interaction with various peptides presented by HLA‐E. In addition, peptides presented by the HLA‐E molecule engage differentially the NKG2A receptor and thereby influence inhibition of this immune checkpoint molecule.

**FIGURE 2:**
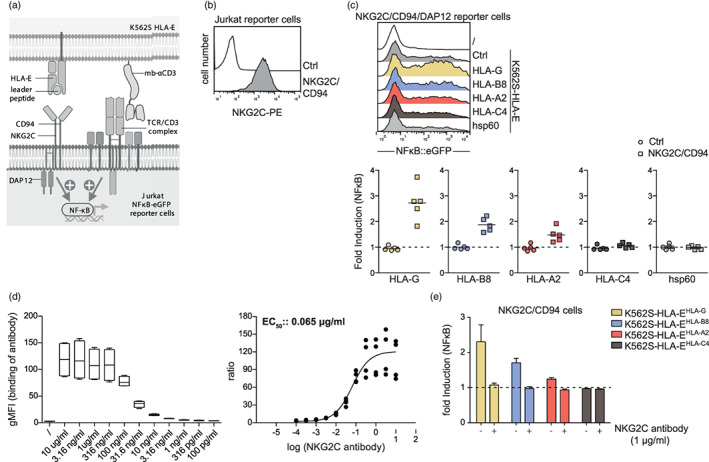
Characterization of NKG2C/CD94/DAP12 function in a reporter cell line. (a) Schematic showing the interaction between NKG2C and the TCR/CD3 complex on the Jurkat reporter cells with HLA‐E‐peptide complexes and membrane‐bound anti‐CD3‐scFv on the T cell stimulator cells (K562S). (b) Cell surface expression of NKG2C on control and NKG2C/CD94/DAP12 expressing reporter cells. (c) Upper panel; histograms of one representative experiment of NKG2C/CD94/DAP12 reporter cells stimulated with control K562S and K562S‐HLA‐E^HLA‐G^, K562S‐HLA‐E^HLA‐B8^, K562S‐HLA‐E^HLA‐A2^, K562S‐HLA‐E^HLA‐C4^ and K562S‐HLA‐E^hsp60^ is depicted. NFκB‐eGFP expression was measured via flow cytometry. Lower panel; control and NKG2C/CD94 expressing reporter cells were co‐cultured with the different HLA‐E‐peptide K562S cells. Reporter activation is shown as fold induction (gMFI of K562S‐HLA‐E^peptide^‐stimulated cells/gMFI control K562S‐stimulated cells). Results of 5 independent experiments performed in duplicates are depicted. (d) NKG2C expressing cells were incubated with increasing concentrations of an NKG2C antibody (clone 134522) ranging from 100 pg/ml to 10 μg/ml. Binding was detected with an APC‐conjugated goat anti‐mouse antibody. (e) NKG2C expressing reporter cells were stimulated with control K562S and K562S‐HLA‐E^HLA‐G^, K562S‐HLA‐E^HLA‐B8^, K562S‐HLA‐E^HLA‐A2^ and K562S‐HLA‐E^HLA‐C4^ in the presence or absence of an NKG2C antibody (1 μg/ml). Reporter activation is shown as fold induction (gMFI of K562S‐HLA‐E^peptide^‐stimulated cells/gMFI control K562S‐stimulated cells). Results of two independent experiments performed in duplicates are depicted.

The counterpart of the inhibitory receptor NKG2A is the activating molecule NKG2C, which also forms a heterodimer with CD94 and associates with the signalling adapter DAP12. To assess the interaction of this receptor with HLA‐E‐peptide complexes at a functional level, we generated reporter cells expressing NKG2C/CD94/DAP12 and co‐cultured the cells with control K562S and K562S expressing peptide‐HLA‐E complexes (Figure 2a–c). We observed a strong increase of NFκB gene expression in NKG2C reporter cells when they engage HLA‐E^HLA‐G^, whereas in the presence of HLA‐E^HLA‐C4^ only a weak stimulatory effect was detected. HLA‐E^HLA‐A2^ and HLA‐E^HLA‐B8^ induced moderate reporter activation via NKG2C, and HLA‐E^hsp60^ failed to functionally engage NKG2C (Figure 2c). In line with our functional data, we observed that Fc‐tagged HLA‐E molecules presenting HLA‐G leader peptide were engaging NKG2C/CD94 expressing cells whereas binding of HLA‐E^HLA‐C4^‐Fc and HLA‐E^HSP60^‐Fc was not detectable (Figure S2). These data demonstrate that NKG2C/CD94 engages HLA‐E‐peptide complexes with a lower affinity than the inhibitory counterpart NKG2A/CD94 [23]. A monoclonal NKG2C antibody (clone 134 522) strongly bound to reporter cells expressing NKG2A/CD94 (Figure 2d). Moreover, this antibody completely blocked the activation via K562S‐HLA‐E^HLA‐G^, K562S‐HLA‐E^HLA‐A2^ or K562S‐HLA‐E^HLA‐B8^ complexes (Figure 2e).

**FIGURE 3:**
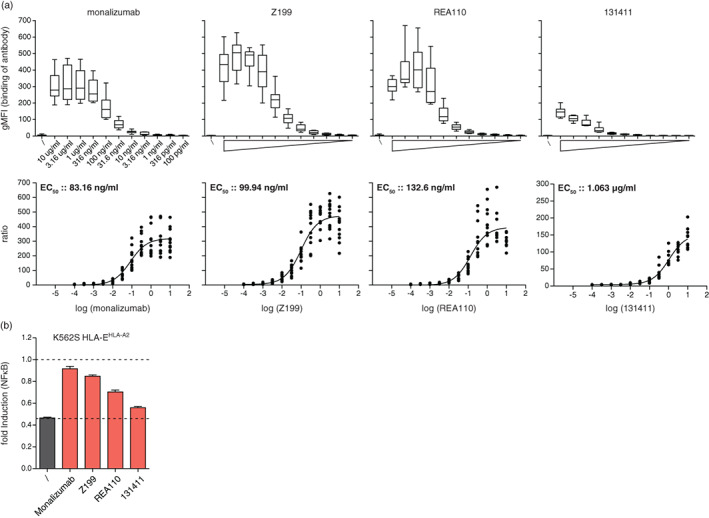
Evaluation of NKG2A antibodies in their binding and blocking capacities. (a) The EC_50_ values were determined for the therapeutic NKG2A antibody monalizumab, as well as for the monoclonal antibodies clone Z199, clone REA110 and clone 131411. NKG2A expressing reporter cells were incubated with increasing concentrations ranging from 100 pg/ml to 10 μg/ml of the depicted antibodies. Antibody binding was detected with an APC‐conjugated anti‐human and anti‐mouse IgG antibody, respectively. EC_50_ was calculated from six experiments performed in duplicate. (b) NKG2A expressing reporter cells were stimulated with control K562S and K562S‐HLA‐E^HLA‐A2^ in the presence or absence of monalizumab, Z199, REA110 and 131411 (all 1 μg/ml). Reporter activation is shown as fold induction (gMFI of K562S‐HLA‐E^HLA‐A2^ stimulated cells/gMFI control K562S‐stimulated cells). Results are shown of four (monalizumab) and two (Z199, REA110 and 131411) independent experiments performed in duplicates.

NKG2A represents a new target to enhance antitumour immunity and clinical trials with monalizumab, a novel ICI has yielded promising results [4, 33]. We analysed the binding of monalizumab and three additional mAb (clones Z199 [mouse], REA110 [human] and 131411 [mouse]) to cells expressing NKG2A/CD94. Strong binding was observed for monalizumab, Z199 and REA110 antibodies, while 131411 resulted in weak binding signals (Figure 3a). EC_50_ values from the binding signals were calculated and yielded values that were in a similar range for monalizumab (83.16 ng/ml), Z199 (99.94 ng/ml) and REA100 (132.6 ng/ml) (Figure 3a). The EC_50_ value measured for clone 131411 was 1.063 μg/ml. Next, we used our reporter platform to analyse the capacity of these antibodies to block the inhibition of reporter gene activation mediated by NKG2A/CD94–HLA‐E^HLA‐A2^ interaction. NKG2A/CD94 reporter cells were co‐cultured with K562S‐HLA‐E^HLA‐A2^ in the presence or absence of the respective NKG2A antibodies. While monalizumab and Z199 were very efficient in blocking the NKG2A and HLA‐E ^HLA‐A2^ interaction, the monoclonal NKG2A antibody REA110 was less effective to revert NKG2A inhibition (Figure 3b). Clone 131411 demonstrated very weakly blocking capacities.

**FIGURE 4:**
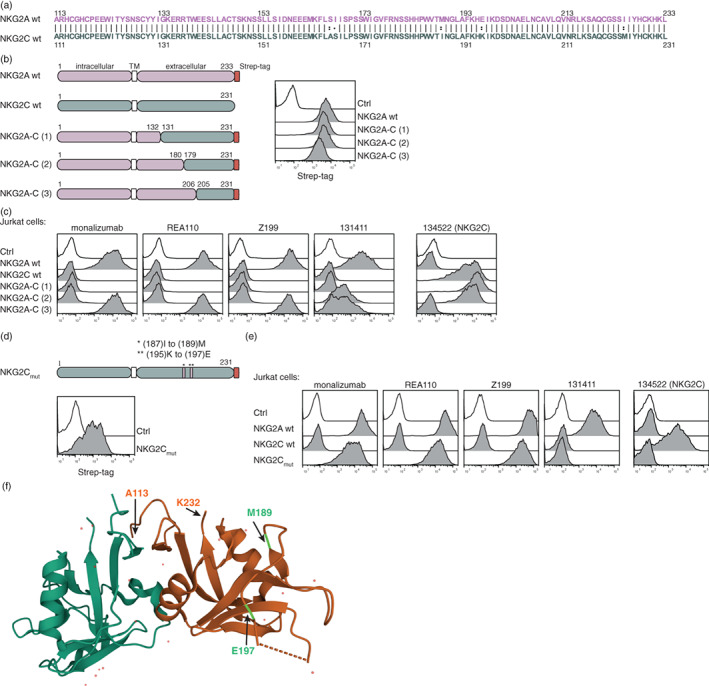
Identification of the epitopes of NKG2A antibodies. (a) Sequence alignment of NKG2A (A113 to L233) and NKG2C (A111 to L231). (b) Schematic representations of wild‐type NKG2A and NKG2C, as well as chimeric constructs of NKG2A‐C (#1, #2, #3), are depicted. Cell surface expression of the molecules was determined by using a Strep‐tag antibody. (c) Jurkat cells expressing the indicated molecules were incubated with the following NKG2A antibodies monalizumab, REA110, Z199, 131411, and a NKG2C antibody (134522), respectively (open histogram: the indicated antibodies on control cells). (d) Schematic representation of a mutated NKG2C construct (NKG2C_mut_) and its cell surface expression detected with a Strep‐tag antibody. (e) Flow cytometric analysis of the depicted antibodies on cells expressing the NKG2C_mut_ molecule (open histogram: the indicated antibodies on control cells). (f) Crystal structure of NKG2A (orange) and CD94 (green) (PDB ID code 3BDW). Positions A113, M189 (light green), E197 (light green) and K232 are highlighted with arrows [35]. One representative experiment is shown (b–e).

NKG2A and NKG2C are type 2 membrane receptors, which share 95% sequence identity, and differ by only a few amino acids in their extracellular domain [34] (Figure 4a). The high similarity between these two receptors could potentially pose a challenge for the development of antibodies that efficiently bind and block the NKG2A, but not the NKG2C receptor. To identify the epitopes of the four NKG2A antibodies, we designed three chimeric constructs harbouring N‐terminal regions of NKG2A fused to NKG2C and a Strep‐tag located at the C‐terminus. These constructs were introduced into our reporter cells and staining with a Strep‐tag antibody confirmed high surface expression of the chimeric proteins (Figure 4b). As expected monalizumab, clone Z199, clone REA110 and clone 131411 were all binding to NKG2A/CD94 and not to NKG2C/CD94, while the NKG2C antibody (clone 134522) specifically reacted with NKG2C/CD94 (Figure 4c). The three blocking antibodies bound strongly to cells expressing NKG2A/C construct #3 harbouring NKG2A residues 1–206, whereas the other two constructs were not bound. By contrast, the NKG2A clone 131411, which is only weakly blocking the interaction between NKG2A and HLA‐E^HLA‐A2^ molecule, was also reacting with cells expressing NKG2A/C construct #2, harbouring NKG2A residues 1–180 (Figure 4c). This indicates that the NKG2A sequence 132–180 might contain the epitope of clone 131411, whereas the epitope of the NKG2A antibodies with strong blocking capacities might be located between amino acids 181 and 206 of the NKG2A molecule. This region only differs from NKG2C by two amino acids (M189/I187 and E197/K195). Therefore, we generated and expressed a mutated version of NKG2C (NKG2C_mut_) by exchanging two amino acids (I187M and K197E) of the wild‐type NKG2C molecule (Figure 4d). Monalizumab, clone Z199 and clone REA110 are strongly bound to this construct demonstrating that 189M and 197E critically contribute to the interaction of blocking antibodies such as monalizumab with NKG2A (Figure 4e). Interestingly, the I187M and K197E mutations completely abrogated the binding of the anti‐NKG2C mAb clone 134522 (to NKG2C_mut_) indicating that blocking antibodies to NKG2C and NKG2A recognize corresponding regions on their antigens (Figure 4e). The location of the two amino acids M189 and E197 in the crystal structure of the NKG2A/CD94 complex (PDB ID code 3BDW) is depicted in Figure 4f [35].

**FIGURE 5:**
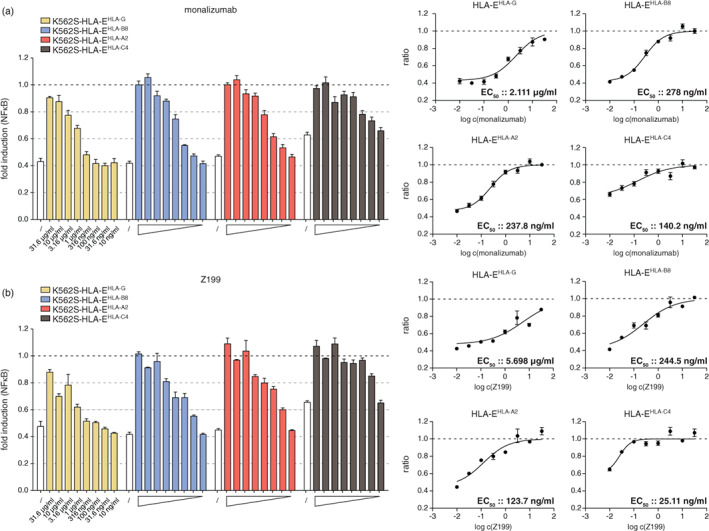
Assessment of functional EC_50_ values of NKG2A blocking antibodies monalizumab and Z199. NKG2A/CD94 expressing reporter cells were stimulated with control K562S, K562S‐HLA‐E^HLA‐G^, K562S‐HLA‐E^HLA‐B8^, K562S‐HLA‐E^HLA‐A2^ and K562S‐HLA‐E^HLA‐C4^ for 24 h in the presence of monalizumab (a) and Z199 (b) at concentrations ranging from 31.6 μg/ml to 10 ng/ml. White bars represent NKG2A inhibition in the absence of an antibody. Results are depicted of three (monalizumab) and two (Z199) independent experiments performed in duplicate. Left panels; data are normalized to control K562S cells in the absence or presence of the indicated antibody concentrations. Right panels; inhibition curves and half‐maximum effective concentrations (EC_50_) were calculated for the NKG2A antibodies from normalized data.

As peptides presented by the HLA‐E molecule determine the interaction with target cells, we assessed the functional efficacy (EC_50_ values) of the four NKG2A antibodies in blocking NKG2A inhibition to peptide‐HLA‐E complexes. Therefore, we co‐cultured NKG2A expressing reporter cells with K562S control, K562S‐HLA‐E^HLA‐G^, K562S‐HLA‐E^HLA‐B8^, K562S‐HLA‐E^HLA‐A2^ and K562S‐HLA‐E^HLA‐C4^ in the presence or absence of monalizumab, Z199 and REA110 at concentrations ranging from 10 ng/ml to 31.6 μg/ml (Figures 5 and S3). For monalizumab and Z199, we observed an increase in reporter gene expression in a dose‐dependent manner when stimulated with different peptide‐HLA‐E complexes. NKG2A inhibition was completely abolished at high concentrations of these antibodies in co‐culture experiments with HLA‐E presenting leader peptides of HLA‐C4, HLA‐A2 and HLA‐B8, while this was not the case for the HLA‐G leader peptide (Figure 5a,b, left panel). The EC_50_ values calculated for monalizumab were 278 ng/ml for HLA‐B8, 237.8 ng/ml for HLA‐A2 and 140.2 ng/ml for HLA‐C4, while the antibody was less effective in blocking HLA‐G presentation with a value of 2.111 μg/ml (Figure 5a, right panel). Similar results were obtained for Z199 with 244.5 ng/ml for peptide HLA‐B8, 123.7 ng/ml for HLA‐A2, 25.11 ng/ml for HLA‐C4 and 5.698 μg/ml for HLA‐G (Figure 5b, right panel). Weak blocking capacities were observed for the NKG2A antibody REA110 and 131411, where total blocking was not accomplished (Figure S3). The EC_50_ values and the 95% CI of monalizumab and Z199 from the functional assays are summarized in Table 1. The higher binding capacities of monalizumab and Z199 in comparison to REA110 and also 131411 is reflecting the functional efficiency in blocking the interaction between NKG2A and peptide‐HLA‐E complexes. To further assess the blocking capacities of monalizumab, we performed a cell conjugation assay that is based on fluorescent cells specifically K562‐NKG2A/CD94 expressing GFP and RFP expressing K562 cells expressing HLA‐E^peptide^ complexes. The results of cell–cell interaction assays pointed to a poor capacity of NKG2A antibodies to block the interaction between NKG2A and HLA‐E^HLA‐G^ (Figure S4). This was also the case if the ligand HLA‐E is blocked with an HLA‐E antibody (3D12) (Figure S5). Taken together, these results highlight that the peptides complexed with HLA‐E impact the blocking capability of NKG2A‐antibodies.

**TABLE 1:** EC_50_ values and the 95% confidence intervals (CI) were determined for monalizumab and Z199 for their ability to block the HLA‐E^peptide^/NKG2A interaction in a functional assay.

	Functional EC_50_ values		
Mab	Peptide‐HLA‐E complex	EC_50_ (ng/ml)	95% CI
Monalizumab	HLA‐E^HLA‐G^	2111	1560–2855
	HLA‐E^HLA‐B8^	278	224.3–344.5
	HLA‐E^HLA‐A2^	237.8	187–302.5
	HLA‐E^HLA‐C4^	140.2	77.73–252.7
Z199	HLA‐E^HLA‐G^	5698	3311–9804
	HLA‐E^HLA‐B8^	244.5	162.6–367.6
	HLA‐E^HLA‐A2^	123.7	77.31–197.9
	HLA‐E^HLA‐C4^	25.11	17.77–35.49

*Note*: Data from three (monalizumab) and two (Z199) independent experiments performed in duplicate were used to calculate the EC_50_ values.

**FIGURE 6:**
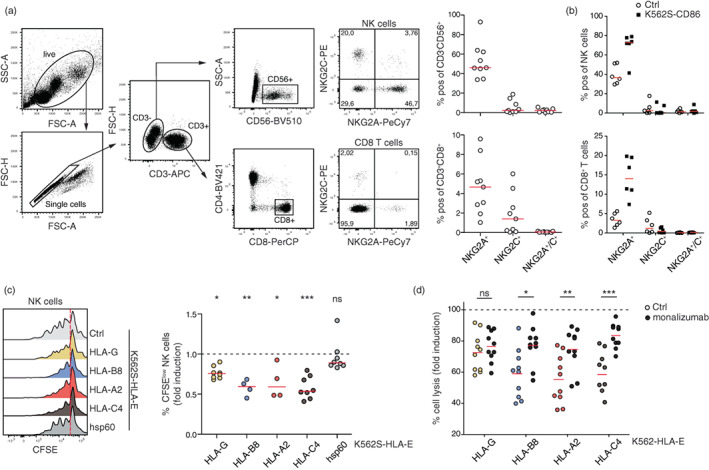
NKG2A expression on CD8^+^ T cells and natural killer (NK) cells. (a) Left panel; representative gating of NKG2A and NKG2C on NK cells and CD8^+^ T cells from freshly isolated PBMCs is shown. Right panel; expression of NKG2A and NKG2C on CD8^+^ T cells and NK cells; each dot represents one donor. Median is shown (*n* = 10). (b) Freshly isolated PBMCs were stimulated with K562S cells expressing CD86 for 5 days. NKG2A and NKG2C expression was assessed on NK and CD8^+^ T cells. Each dot represents one donor. Median is shown (*n* = 6). (c) PBMCs were co‐cultured for 5 days with the indicated K562S cell lines and proliferation (CSFE^low^) was analysed for NK cells. Left panel; one representative experiment is shown. Right panel; data from several donors are depicted (HLA‐G and HLA‐C4 [*n* = 8]; HLA‐B8 and HLA‐A2 [*n* = 4]). Each data point represents the mean of triplicates of one donor. NK cell proliferation induced upon stimulation with K562S expressing the indicated HLA‐E‐complexes is normalized to the NK cell proliferation induced by control K562S cells (% of CFSE^low^ of K562S‐HLA‐E^peptide^ stimulated NK cells/% of CFSE^low^ control K562S‐stimulated NK cells). For statistical evaluation, a one‐way ANOVA followed by Dunn's multiple comparison test was performed (***p ≤ 0.001; **p ≤ 0.01; *p ≤ 0.05; ^ns^ *p* > 0.05). (d) Lysis of the indicated target cells upon 4 h co‐culture with PBMCs pre‐stimulated for 7–10 days in the presence of IL‐2 and IL‐15 to induce NK cell expansion. Effector: target cell ratio was 10:1 and monalizumab was used at a final concentration of 5 μg/ml. Data were normalized to control K562 cells (control K562 = 100% cell lysis). Median is shown (*n* = 10). For statistical evaluation, a two‐way ANOVA followed by Bonferroni post hoc test was performed (***p ≤ 0.001; **p ≤ 0.01; *p ≤ 0.05; ^ns^ *p* > 0.05).

The inhibitory receptor NKG2A was described to be mainly expressed on NK cells, but recently it was shown to also play a crucial role on CD8^+^ T cells during the antitumour immune response [4, 9]. When we assessed the expression of NKG2A and also NKG2C on NK cells and CD8^+^ T cells from healthy donors, we detected NKG2A surface expression on a large proportion of NK cells (≈40%), whereas only about 3% of CD8^+^ T cells stained positive for NKG2A. NKG2C was only present in around 8% of NK cells and 2% of CD8^+^ T cells. Only a few NK cells and almost no CD8^+^ T cells co‐expressed NKG2A/NKG2C (Figure 6a). Upon in vitro stimulation of PBMCs via the TCR complex and the costimulatory molecule CD28 by using T cell stimulator cells expressing CD86 (K562S‐CD86), we observed an upregulation of NKG2A cell surface expression on both cell populations (NK cells and CD8^+^ T cells) (Figure 6b). Data obtained with our reporter system indicate that the capacity of HLA‐E‐peptide complexes to prevent NK‐mediated lysis might depend on the peptides presented by HLA‐E. To test this, we co‐cultured PBMCs from healthy donors with control K562 stimulator cells and K562S expressing different HLA‐E‐peptide complexes and assessed the proliferation of NK cells. As expected, compared to control K562S cells and K562S‐HLA‐E^hsp60^, which cannot functionally engage NKG2A, the proliferation of NK cells was strongly reduced in the presence of K562S cells expressing the NKG2A ligands HLA‐E^HLA‐G^, HLA‐E^HLA‐B8^, HLA‐E^HLA‐A2^ or HLA‐E^HLA‐C4^. Interestingly, HLA‐E^HLA‐G^ complexes, which mediated the strongest inhibition in NKG2A expressing reporter cells, did not induce a stronger inhibition of NK cell proliferation than HLA‐E^HLA‐B8^, HLA‐E^HLA‐A2^ and HLA‐E^HLA‐C4^ (Figures 6c and S6A). The ultimate goal of checkpoint inhibitors like monalizumab is to promote the elimination of tumour cells by effector cells. Consequently, we analysed the efficacy of monalizumab to enhance NK cell‐mediated killing of target cells expressing different HLA‐E‐peptide complexes. PBMCs were stimulated for 7–10 days with IL‐2 and IL‐15 to induce activation and proliferation of NK cells. Subsequently, NK cell expansion cultures were co‐cultured with control K562 cells or K562 cells expressing different HLA‐E‐peptide complexes. Compared to control K562 cells or K562‐HLA‐E^hsp60^, K562 cells expressing HLA‐E^HLA‐G^, HLA‐E^HLA‐B8^, HLA‐E^HLA‐A2^ or HLA‐E^HLA‐C4^ cells were significantly less susceptible to NK‐mediated killing (Figures 6d and S6B). Monalizumab greatly increased killing of target cells presenting HLA‐E^HLA‐B8^, HLA‐E^HLA‐A2^ or HLA‐E^HLA‐C4^. HLA‐E^HLA‐G^ complexes appeared to confer less protection than HLA‐E molecules presenting other MHC class I leader peptides and the presence of monalizumab did only marginally increase the killing of target cells expressing HLA‐E^HLA‐G^ (Figures 6d and S6B).

## DISCUSSION

Blocking inhibitory receptors expressed on T cells such as PD‐1 and CTLA‐4 has been a breakthrough in the treatment of cancer [36, 37]. Consequently, there is great interest in targeting additional inhibitory receptors such as LAG‐3, TIM‐3 or TIGIT to enhance anticancer immunity [2]. NKG2A is another emerging immunotherapy target whose blockade could promote both T cell‐ and NK cell‐mediated anti‐cancer activity. Recent studies have demonstrated that NKG2A blockade can enhance the efficacy of peptide vaccination in different mouse solid tumour models [9]. In vivo CRISPR screening demonstrated that the NKG2A pathway interfered with immunotherapy corroborating the potential of NKG2A as a therapeutic target [38]. Andre et al. have shown that monalizumab, a blocking NKG2A antibody enhanced NK cell activity against tumour cells. Moreover, this antibody was effective in augmenting CD8 T cell function when combined with PD‐1 inhibitors indicating that established ICI therapies can be improved by co‐blockade of NKG2A.

However, NKG2A function is complex since it critically depends on the peptides presented by its sole ligand, the MHC class 1b member HLA‐E in humans, or Qa‐1^b^ in mice. Whereas cell line models have been described for inhibitory costimulatory receptors such as PD‐1 or CTLA‐4 and were used to evaluate ICIs targeting these molecules, to date reductionist systems to study NKG2A mediated inhibition have not been available [27, 39]. Here, we used a transcriptional reporter system based on the Jurkat T cell line to study how peptide presentation by HLA‐E impacts the NKG2A signalling and the efficacy of antibodies targeting this receptor. SCT comprising the ectodomain of the HLA‐E heavy chain, β2‐microglobulin and a disulfide‐trapped leader sequence were used as NKG2A ligands. This approach avoids problems associated with exogenous peptide loading such as variable surface density of HLA‐E molecules and exchange of peptides of interest with other HLA‐E peptide ligands [25]. In our study, we have focused on the well‐characterized leader sequences of HLA‐G, HLA‐B8, HLA‐A2 and HLA‐C4 and a peptide derived from hsp60. When we co‐cultured NKG2A/CD94 expressing reporter cells with K562‐based stimulator cells displaying HLA‐E complexed with 9‐mer peptides derived for HLA‐G, we observed a strong inhibition of reporter activation, while a corresponding peptide derived for the leader sequence of HLA‐C4 was less effective in engaging the inhibitory NKG2A receptor. This is in line with earlier reports showing that the 9‐mer peptide from HLA‐G (VMAPRTLFL) has a high affinity to NKG2A in comparison to the HLA‐Cw7‐derived peptide VMAPRTLLL [23, 34]. Our results indicate that in addition to the well‐described HLA‐G peptide, other signal peptides like HLA‐B8 and HLA‐A2 complexed with HLA‐E can also strongly engage NKG2A.

NKG2A/C chimeric receptors were used to identify the epitopes of monalizumab and other NKG2A antibodies. Monalizumab and clone Z199, which both efficiently blocked NKG2A inhibition, were found to bind a region on NKG2A that differs only by two amino acids (M189/E197) from NKG2C. In addition, we detected that grafting these two amino acids onto the NKG2C sequence was sufficient to confer strong binding of both NKG2A blocking antibodies. The high similarity of the extracellular parts of NKG2A and its activating counterpart NKG2C could potentially put constraints on the development of efficient blocking antibodies targeting NKG2A. Importantly, we found that the blocking capacity of monalizumab and additional NKG2A antibodies greatly depended on the HLA‐E‐peptide complex. The EC_50_ values of monalizumab calculated for HLA‐E^HLA‐C4^ in comparison to HLA‐E displaying the HLA‐G derived signal peptide were more than 15‐fold lower. Monalizumab was efficient in blocking NKG2A mediated inhibition by HLA‐E complexed with signal peptides derived from HLA‐B8, HLA‐A2 and HLA‐C4. Nevertheless, also in the presence of these ligands, the functional EC_50_ values determined for monalizumab were considerably higher compared to clinically used ICIs binding PD‐1 and especially PD‐L1 [39].

Insufficient blockade by NKG2A antibodies was described in a recent study by Kamiya et al. who found that abrogation of NKG2A surface expression by intracellular retention was much more effective in reinstalling cytotoxicity towards HLA‐E expressing target cells than blockade with NKG2A mAb Z199 [40]. We found this antibody to be highly effective in functionally blocking HLA‐E^HLA‐C4^ complexes, whereas high concentrations of this antibody were required to block the HLA‐E complex with signal peptides from other MHC class I molecules and especially HLA‐G.

Cytotoxicity experiments with primary NK cells expanded in vitro indicated that the presence of NKG2A ligands reduced their killing activities and protected the target cells. Interestingly, we observed that HLA‐E^HLA‐G^ complexes were less effective in this setting than HLA‐E molecules presenting the HLA‐C4 signal peptide. Increased cytotoxicity upon engagement of HLA‐E^HLA‐G^ by the activating NKC2C receptor on a small portion of NK cells is a likely explanation of the lower overall protective effect of HLA‐E^HLA‐G^ complexes. Monalizumab partially reversed protection and led to NK cell‐induced lysis upon engagement with HLA‐E displaying signal sequences of HLA‐A2, HLA‐B8 and HLA‐C4. However, the antibody was less effective in increasing the cytotoxicity towards target cells expressing HLA‐E^HLA‐G^ complexes. This aligns with a study by Krijgsman et al. that suggests that human malignant cells can induce de novo expression and presentation of HLA‐G molecules, which could be potentially important for the treatment of tumour patients with monalizumab [41].

Our data highlight the importance of MHC class I leader sequences presented by HLA‐E for NKG2A‐mediated inhibition of NK cells. We show for the first time that peptides displayed by HLA‐E strongly influence the blocking capacity of NKG2A antibodies. This has important implications for tumour therapy since NKG2A is considered an emerging target in cancer immunotherapy and the NKG2A antibody monalizumab is already evaluated in several clinical trials for the treatment of solid tumours. Our study indicates that the expression and presentation of molecules like HLA‐G, which promote the formation of high‐affinity NKG2A ligands could not only represent an immune evasion strategy for tumours, but also a potential resistance mechanism in therapies with ICIs targeting NKG2A [41].

## AUTHOR CONTRIBUTIONS

Claire Battin performed the majority of experiments, supervised experimental work, designed the study and wrote the manuscript. Gabriel Kaufmann performed experiments. Judith Leitner, Joshua Tobias, Ursula Wiedermann, Alexander Rölle and Marten Meyer provided essential reagents. Frank Momburg provided essential reagents and supported study design. Peter Steinberger supervised experimental work, designed the study and wrote the manuscript. All authors critically revised the study.

## CONFLICT OF INTEREST

The authors declare no conflict of interest.

## Supporting information


**FIGURE S1** **Binding of Fc‐tagged/fused HLA‐E molecules to control and NKG2A/CD94 expressing reporter cells.** Cells were incubated with HLA‐E single chain trimer‐Fc molecules at concentrations ranging from 3 ng/mL to 10 μg/mL. Binding was detected with a PE‐conjugated goat anti‐mouse (Fc‐specific) antibody.

**FIGURE S2** **Binding of Fc‐tagged/fused HLA‐E molecules to control and NKG2C/CD94/DAP12 expressing reporter cells.** Cells were incubated with HLA‐E single chain trimer‐Fc molecules at concentrations ranging from 3 ng/mL to 10 μg/mL. Binding was detected with a PE‐conjugated goat anti‐mouse (Fc‐specific) antibody.

**FIGURE S3** **Functional evaluation of NKG2A antibodies REA110 and 131 411 in blocking HLA‐E‐peptide complexes.** NKG2A/CD94 expressing reporter cells were stimulated with control K562S, K562S‐HLA‐E^HLA‐G^, K562S‐HLA‐E^HLA‐B8^, K562S‐HLA‐E^HLA‐A2^ and K562S‐HLA‐E^HLA‐C4^ for 24 h in the presence of REA110 (A) at concentrations ranging from 31.6 μg/mL to 10 ng/mL. 131 411 was co‐cultured with reporter and stimulator cells at concentrations of 10 μg/mL and 1 μg/mL (B). Flow cytometric measurement of NFκB‐eCFP was assessed. Results are depicted for two independent experiments performed in duplicate. Data is normalized to control K562S cells in the absence or presence of the indicated antibody concentrations.

**FIGURE S4** **Interaction of NKG2A with its ligand HLA‐E** ^**peptide**^ **in a cell–cell conjugation assay** A, B) K562 NKG2A/CD94 expressing eGFP and K562 HLA‐E^HLA‐G or HLA‐C4^ expressing RFP were incubated for 2 h in the presence or absence of 5 and 1 μg/mL monalizumab. Conjugate formation (% double positive) expressing the two fluorescent proteins (eGFP/eCFP) was assessed via flow cytometry. One representative experiment is shown. One representative experiment is shown (A and B).

**FIGURE S5** **Evaluation of blocking NKG2A/HLA‐E interaction with a HLA‐E antibody** NKG2A/CD94 expressing reporter cells were stimulated with control K562S, K562S‐HLA‐E^HLA‐G^, K562S‐HLA‐E^HLA‐A2^ and K562S‐HLA‐E^HLA‐C4^ for 24 h in the presence of a HLA‐E antibody (3D12; 5 μg/mL). Flow cytometric measurement of NFκB‐eCFP was assessed. Results are depicted for two independent experiments performed in duplicate.

**FIGURE S6** A) PBMCs were left unstimulated or co‐cultured for 5 days with the indicated K562S cell lines and proliferation (CSFE^low^) was analysed for NK cells. Raw data from several donors are depicted (K562 control, HLA‐G and HLA‐C4 (n = 8); HLA‐B8 and HLA‐A2 (n = 4)). B) Lysis of the indicated target cells (K562 cells) upon 4 h co‐culture with NK cell expansion cultures in the presence or absence of monalizumab (5 μg/mL) (effector: target ratio 10:1). Median is shown (n = 8).

## Data Availability

Data available on request from the authors.
